# Low perceived social support in mothers during pregnancy and early childhood; associations with anxiety and ADHD symptoms in children at 3 and 8 years

**DOI:** 10.1007/s00127-024-02792-1

**Published:** 2024-11-06

**Authors:** Christine Baalsrud Ingeborgrud, Beate Oerbeck, Svein Friis, Are Hugo Pripp, Heidi Aase, Guido Biele, Søren Dalsgaard, Kristin Romvig Overgaard

**Affiliations:** 1 Institute of Clinical Medicine, Division of Mental Health and Addiction, Child and Adolescent Psychiatry Unit, University of Oslo, P.O. Box 1039, Blindern, Oslo, 0315 Norway; 2 Division of Mental Health and Addiction, Oslo University Hospital, Oslo, Norway; 3 Oslo Centre of Biostatistics and Epidemiology, Oslo University Hospital, Oslo, Norway; 4 Department of Child Health and Development, Norwegian Institute of Public Health, Oslo, Norway; 5 Child and Adolescent Mental Health Services, Copenhagen University Hospital, Copenhagen, Denmark; 6 Department of Clinical Medicine, University of Copenhagen, Copenhagen, Denmark

**Keywords:** Social support, Child, Anxiety, ADHD

## Abstract

**Purpose:**

Low perceived social support is associated with adverse effects on maternal mental health, and often coexists with other risk factors for offspring anxiety and attention-deficit/hyperactivity disorder (ADHD). We aimed to investigate whether low maternal social support during pregnancy and early childhood predicted anxiety and ADHD symptoms in children at ages 3.5 and 8 years.

**Methods:**

This study is part of the longitudinal, population-based Norwegian Mother, Father, and Child Cohort Study. Mothers were queried about perceived social support twice during pregnancy, and again at child ages 18 months and 3 years. They were interviewed about their children’s symptoms of anxiety and ADHD at 3.5 years. At 8 years (*n* = 781), the Child Symptom Inventory-4 was used to identify children who fulfilled the criteria for anxiety disorders and ADHD. Logistic regression models estimated the risk of child anxiety and ADHD, depending on maternal social support.

**Results:**

Low maternal social support predicted child anxiety symptoms at both ages 3.5 and 8 years as well as ADHD symptoms at 8 years. When including other maternal stressors and child risk factors, low maternal social support remained a significant predictor for child anxiety symptoms at 3.5 years, and there was a trend towards also predicting child anxiety and ADHD symptoms at 8 years.

**Conclusion:**

The associations between low maternal social support and child symptoms of anxiety and ADHD found in the present study, suggest that focusing on mothers with low social support may hold significance for child symptoms years later.

**Supplementary Information:**

The online version contains supplementary material available at 10.1007/s00127-024-02792-1.

## Introduction

Social support, defined as the extent to which an individual has access to, or perceives having access to, assistance and resources provided by others in their social network [1], has been linked to mental health, and may protect against the negative effects of stress [2–4]. However, received social support is part of a complex interplay with other health determinants, all of which moderate genetic and environmental vulnerabilities and resilience in the face of adversity [5]. Low perceived social support in mothers has been associated with maternal anxiety and depression in pregnancy [6] and from child age 18 months until 13 years [7], maternal attention-deficit/hyperactivity disorder (ADHD) [8], and living without a partner [9]. These maternal stressors are also known risk factors for mental disorders in offspring [10–12]. Previously, we found that maternal anxiety and/or depression during pregnancy and before child age 5 years, as well as maternal ADHD, were associated with an increased risk of child anxiety at 8 years [12].

A systematic review suggested that social capital, including low social support, was linked to mental health and behavioral problems in children and adolescents [13]. However, the review included studies investigating a range of different aspects of social support, mainly focusing on the children’s social relationships, and did not differentiate between various child mental health and behavioral problems as outcomes. Furthermore, as acknowledged by the authors, the cross-sectional design of most of the studies in the review prevented them from drawing firm conclusions about the direction of associations.

As noted by Yan 2023 [14], there is a paucity of research on the impact of parents’ perceived social support on children’s mental health and how environmental family factors affect young children’s symptoms. The same study, with children age 3–6 years, found that the greater the parents’ perceived social support, the less likely young children were to experience mental health problems (measured by The Strengths and Difficulties Questionnaire) [14]. However, their study was limited by its cross-sectional design and did not distinguish between mothers’ and fathers’ reports on perceived social support. Generally, there is a lack of studies on paternal social support and its influence on child symptoms.

A recent Japanese study found that maternal social isolation one year after delivery was associated with an elevated risk of internalizing (including anxiety) as well as externalizing problems (including ADHD) in four-year-olds [15]. However, the authors underlined the limited generalizability of the study, as it was conducted in just one of the numerous prefectures in Japan. No studies have prospectively examined the association between mothers’ low social support and subsequent anxiety and ADHD symptoms in their children at multiple time points, or in children as old as 8 years.

In this prospective, longitudinal cohort study, our objectives were threefold. First, we aimed to delineate associations between low social support and other maternal stressors. We hypothesized that a greater proportion of mothers with low social support would experience additional stressors (such as anxiety/depression, ADHD, living without a partner or having a partner reporting low social support, and/or low education level) compared to their counterparts. Second, we aimed to investigate whether low maternal social support during pregnancy and/or infancy were risk factors for later anxiety and ADHD symptoms in children at 3.5 years and 8 years, respectively. Finally, if low social support was found to predict child anxiety and ADHD symptoms, we aimed to examine whether these associations would be altered by the inclusion of the mentioned maternal stressors.

## Methods

### Participants

The Norwegian Mother, Father, and Child Cohort Study (MoBa) is an ongoing population-based cohort study conducted by the Norwegian Institute of Public Health. From 1999 to 2008, pregnant Norwegian-speaking women undergoing their first ultrasound were enrolled from all over Norway (*n* ∼ 114,500 children; 41% participation rate) [16]. The current paper is part of the ADHD substudy oversampling children with high scores of ADHD-symptoms, using an 11-item screening measure from the 36-month MoBa questionnaire, a procedure previously described in detail [17, 18]. In brief, 80% of the invited participants (*n* = 2,798) had scores ≥ 90th percentile on the ADHD screening items, while the remaining 20% (*n* = 654) were randomly selected from MoBa. 35% agreed to participate, and 1,195 children (mean age 3.5 years) took part in a one-day clinical assessment, including a diagnostic interview with their mothers (except for a handful of participants where fathers were interviewed). A small number withdrew their consent to participate during the study, leaving 1180 participants (52% boys (*n* = 618)). The current study included children with completed parent-reports at ages 3.5 and 8 years (*n* = 781). Supplemental Fig. 1 shows a flowchart for the study enrolment and attrition.

### Measures

#### Social support

In the present study, we used two items available in MoBa, which were self-reported by mothers twice during pregnancy (at weeks 15 and 30) and twice during early childhood (at ages 18 and 36 months) and by fathers at pregnancy week 15. We defined low social support as low perceived support, and/or feelings of loneliness. A non-affirmative response to the question “Do you have anyone other than your husband /partner you can ask for advice in a difficult situation?” was considered indicative of low perceived social support. Loneliness was measured by asking “Do you often feel lonely?”. The response categories “never”, “infrequently”, or “sometimes” were considered typical, while “usually” and “often” were defined as loneliness.

#### Maternal anxiety and depression

Mothers reported their symptoms of anxiety and depression on the eight-item version (SCL-8) of the Hopkin Symptom Checklist (SCL-25) [19] at week 30 during pregnancy, and at child age 6 months, 18 months and 3 years. At week 15 during pregnancy, the five-item version (SCL-5) was used. The SCL-8 consists of four items measuring depressive symptoms (‘Feeling hopeless about the future’, ‘Feeling blue’, ‘Worrying too much about things’, ‘Feeling everything is an effort’) and four measuring anxiety symptoms (‘Feeling fearful’, ‘Nervousness or shakiness inside’, ‘Feeling tense or keyed up’, ‘Suddenly scared for no reason’). The SCL-5 comprises the first three depressive symptoms and the first two anxiety symptoms. Each item is rated on a four-point Likert scale (1–4) as occurring as ‘not at all’, ‘a little’, ‘quite a bit’, or ‘extremely’, resulting in sum scores from 5 to 20 for the SCL-5 and from 8 to 32 for the SCL-8. The average item score was determined by dividing the sum score by the number of items answered. The cut-off was set to ≥2, which is consistent with suggestions for the SCL-5 [20]. The correlation between the SCL-8 and SCL-25 was high (0.94) [21], and the SCL-5 was found to perform almost equally well as the full version [20]. Cronbach’s alpha values ranged from 0.79 to 0.88 for the SCL-5 and SCL-8 [12].

#### Maternal ADHD

At child age 3 years, mothers reported their symptoms of ADHD on the six-item Adult Self-Report Scale (ASRS-6); with four items measuring inattention (IA) and two items measuring hyperactivity–impulsivity (HI), consistent with the DSM-IV criteria [22], and previously validated in the MoBa [23]. The items are scored on a five-point Likert scale (0–4) as occurring as ‘never’, ‘rarely’, ‘sometimes’, ‘often’, or ‘very often’, leading to sum scores ranging from 0 to 24, with scores of ≥14 as the cut-off [24]. Cronbach’s alpha was 0.70 [12].

#### Socioeconomic factors

Childbirth dates were obtained from the Norwegian Medical Birth Registry. The duration of parental education was obtained from the initial MoBa assessment during pregnancy and categorized based on whether the mean education length of the parents was 12 years or more or less than 12 years (equivalent to whether the parents had finished secondary school in Norway or not). Cohabitation status reported (mainly by the mother) at 3 years, as either “living with the father” or “not living with the father”.

### Outcome

#### Preschool symptoms of anxiety and ADHD

Parents were interviewed when the children were 3.5 years using a semi-structured diagnostic interview, the Preschool Age Psychiatric Assessment (PAPA) [25], and symptoms of anxiety and ADHD were scored as “present” or “not present”, respectively, by trained psychology students. Symptoms present during the previous three months were counted and then aggregated into a symptom sum score. In line with the PAPA guidelines, the anxiety sum score included seven symptoms of specific phobia, three symptoms of social phobia, seven symptoms of separation anxiety, and six symptoms of generalized anxiety. Following the algorithm for generalized anxiety, these symptoms were only counted when the criterion ‘worries’ was reported. We conducted thorough analyses to evaluate different thresholds to prevent under- or over-inclusion. Based on statistical assessment, the cut-off for the anxiety group at 3.5 years was set at ≥ 3 symptoms from any of the anxiety subcategories. Consistent with previous studies [17, 18], we used information from the PAPA, and defined ADHD by the DSM-IV-TR criteria [26] with at least six out of nine symptoms of IA and/or HI present.

#### Anxiety and ADHD categories at 8 years

The parents completed the Child Symptom Inventory-4 (CSI-4), a questionnaire with items and algorithms for diagnoses derived from the DSM-IV diagnostic criteria [27]. The CSI-4 has been found to provide reliable and valid measurements, with temporal stability over four years for most symptom categories [28]. The CSI-4 was rated on a four-point Likert scale (0–3) as occurring as ‘never’, ‘sometimes’, ‘often’, or ‘very often’ [27], and was dichotomized as being present or not present, in line with the algorithms in the CSI manual. The diagnostic cut-off scores were set to the minimum number of symptoms necessary for DSM-IV diagnoses of anxiety disorders and ADHD.

Anxiety disorders at 8 years included specific phobia, social phobia, separation anxiety, and generalized anxiety disorder. For specific phobia, the CSI-4 contains one symptom, which we required to be present “often” or “very often”. For social phobia, at least three of four symptoms had to be endorsed to fulfil the criteria. For separation anxiety, at least three of eight symptoms had to be endorsed. For generalized anxiety disorder, eight items are listed, where the modified criteria ‘Over-concerned about abilities in academic, athletic, or social activities’ or ‘Has difficulty controlling worries’ had to be endorsed for the other symptoms to be counted, and at least three symptoms had to be endorsed to be categorized with general anxiety disorder. For ADHD, at least six of either the nine symptoms of IA or the nine symptoms of HI had to be endorsed.

Supplemental Table 1 shows the measurements and number of participants at the different timepoints.

### Ethics

MoBa and the initial data collection were based on a licence from the Norwegian Data Protection Agency and approval from the Regional Committees for Medical and Health Research Ethics. The MoBa cohort is currently regulated by the Norwegian Health Registry Act. The present study was approved by the Regional Committees for Medical and Health Research Ethics (2017/1276).

### Statistics

Statistical analyses were performed using SPSS Statistics for Windows, version 26. *T-* tests were used to compare means, and Pearson chi-square tests were used to compare categorical variables. First, univariable logistic regression analyses were used in unadjusted models to estimate the odds of child anxiety and ADHD symptoms at 3.5 and 8 years, in the presence of low maternal social support as well as other predictors. Second, in the multivariable logistic regression models, we used stepwise elimination, where those predictors that contributed to the model at *p* < .2 were kept in the analysis. The multivariable model included low maternal social support on at least one occasion from pregnancy to child age 3 years, maternal anxiety and/or depression on at least one occasion (also from pregnancy to child age 3 years), maternal ADHD, parental marital status, paternal report of low social support, and parental education. We also checked whether controlling for child symptoms of anxiety and ADHD at 3.5 years, altered the models with anxiety and ADHD categories at 8 years as outcomes. Odds ratios (ORs) and 95% confidence intervals (CIs) were computed. Tests were two-tailed, and significance levels were set at *p* < .05.

## Results

Compared with mothers reporting good social support, mothers reporting low social support at least once had higher mean anxiety/depression (SCL) and ADHD (ASRS) symptom scores themselves at all assessments and a shorter mean education length (Table 1). A greater proportion of mothers with low social support were living without a partner during pregnancy (8.9% vs. 2.5%, *p* = .005) or were separated/divorced in preschool age (11.1% vs. 3.0%, *p <* .001) compared to their counterparts.

**Fig. 1:**
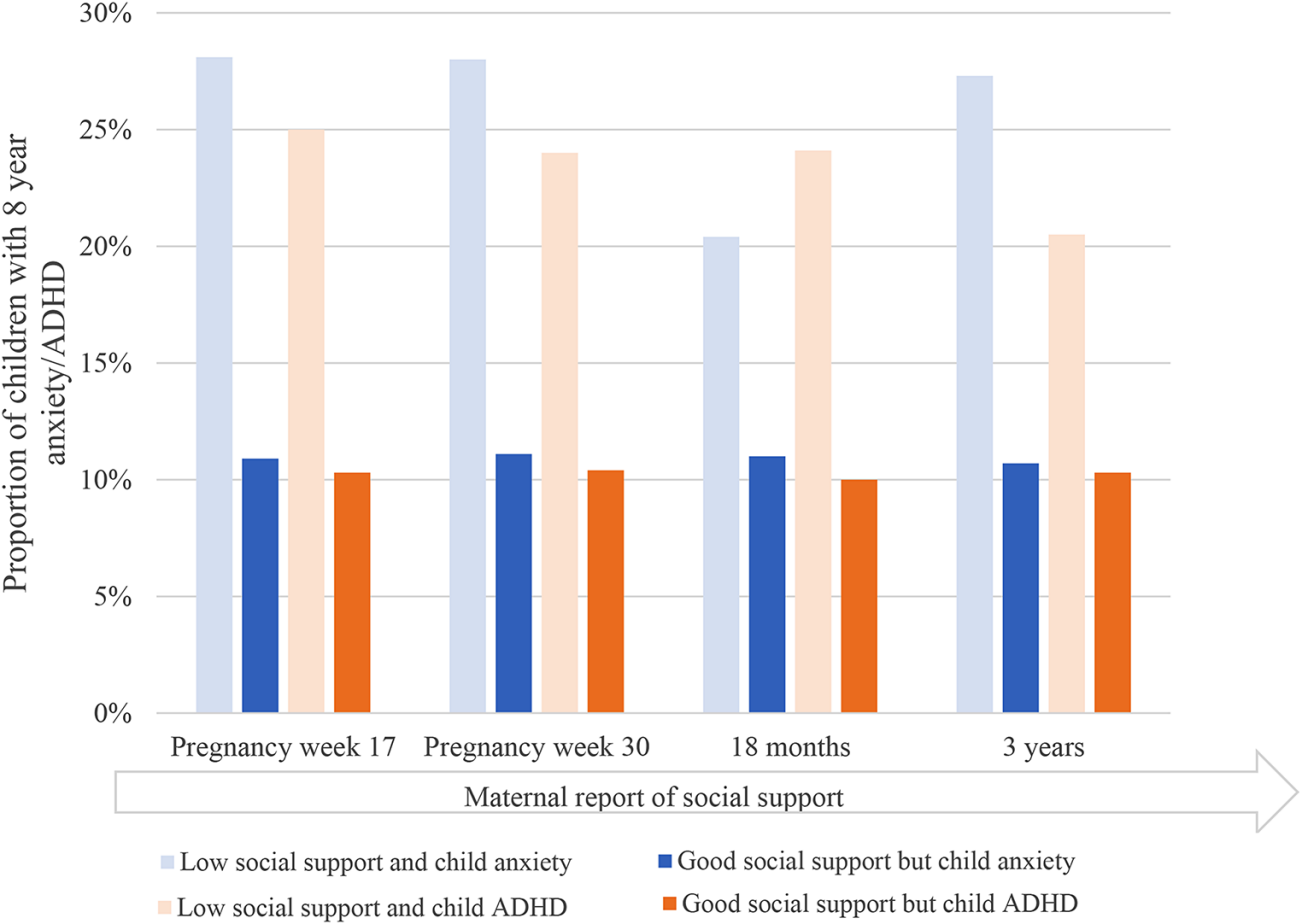
Percentages of children categorized with anxiety disorders or ADHD at the age of 8 years by maternal social support level (low/good) at each assessment point. *Note* P- values for comparisons between the group with low maternal social support and the group with maternal good social support, were < 0.05 at all time points for both child anxiety and ADHD

At each of the four assessments, a significantly greater proportion of children whose mothers reported low social support met the criteria for anxiety and/or ADHD at 8 years compared to children of mothers reporting good social support at the same time point (Fig. 1).

In the univariable regression analyses, low maternal social support predicted child anxiety symptoms at both ages 3.5 and 8 years (Table 2). Low maternal social support was also a significant predictor for child ADHD at 8 years, but not at 3.5 years. Low paternal social support was a significant predictor for child ADHD at 3.5 years but not at 8 years or for child anxiety.

In the multivariable analyses, when controlling for coexistent maternal stressors and child risk factors, low maternal social support remained a significant predictor for child anxiety at 3.5 years (OR = 2.42, *p* = .004) and was just above the threshold for statistical significance for child anxiety and ADHD at 8 years (ORs = 1.92 and 1.98, *p*s = 0.06), respectively (Table 3).

## Discussion

Our main finding was that low maternal social support predicted child anxiety symptoms at both ages 3.5 and 8 years, and child ADHD symptoms at 8 years. When accounting for additional maternal stressors and child risk factors, low maternal social support remained a significant predictor for child anxiety symptoms at 3.5 years. Furthermore, low maternal social support weakly predicted child anxiety and ADHD symptoms at 8 years, though this association was just above the significance threshold.

Consistent with our hypothesis, mothers reporting low social support also experienced other stressors (anxiety/depression, ADHD, lower education, living without a partner) more often. This aligns with the growing body of literature connecting social support to mental health [29]. The associations between social support and depression, anxiety, and ADHD, respectively, are well known in the adult population [6, 30–32].

In line with our hypothesis, low maternal social support statistically predicted child anxiety symptoms at 3.5 and 8 years. When accounting for other maternal stressors and child risk factors, low maternal social support remained a significant predictor for child anxiety symptoms at 3.5 years, and there was also a trend towards significantly predicting child anxiety symptoms at 8 years. The stronger association at age 3.5 years might possibly be explained by the fact that the exposure was measured closer in time to the outcome than at age 8 years. Our finding is in line with a Japanese study of preschoolers, in which maternal isolation was associated with child internalizing problems (anxiety and withdrawal scores) at 4 years [15]. Shared genetic risk factors may account for part of our observations, as previously shown [12]. However, our findings indicate that there is an association between maternal social support and child anxiety symptoms beyond what can be explained by maternal symptoms. It has been suggested that good maternal social support may impact a child’s symptoms indirectly by moderating the impact of parental mental health [33, 34], or by influencing the parenting style [35]. Future studies may aim to investigate whether social support could influence the association between an overprotective parenting style and child anxiety previously found [36, 37]. Furthermore, the child’s interaction with members of the maternal social network may have a direct impact on the child [38]. Wider socialization experiences have been suggested to influence trajectories of anxiety in early childhood [37]. Although complex, our findings and the abovementioned literature suggest that parents who are predisposed to anxiety and withdrawal may transmit these traits to children in families with limited interaction with other adults.

Previously, it was found that childhood ADHD is negatively associated with the social network of parents [39]. In the current study, ADHD symptoms at 8 years were assessed several years after parents reported perceived social support, suggesting that low social support may be one of multiple environmental risk factors associated with ADHD [40]. For families grappling with hyperactivity, impulsivity and inattention, social support may aid parents in enhancing their parenting skills [38]. Furthermore, perceived social support in the form of practical assistance and help with childcare, could be a protective factor in these families [38].

Low perceived social support did not predict child ADHD symptoms at 3.5 years. The continuity of ADHD symptoms was only moderate from 3.5 to 8 years [41], suggesting that it may be too early to apply the ADHD diagnostic symptom criteria at 3.5 years. However, the higher estimated heritability for ADHD than for anxiety disorders [42, 43] may contribute to explaining why environmental factors are less significant for ADHD at an early age.

Low paternal social support, reported at week 15 in pregnancy, predicted child ADHD symptoms at 3.5 years, suggesting that this may be one of a variety of psychosocial factors correlated with child ADHD [40]. A previous study on social support and child symptoms including fathers has not distinguished between mothers’ and fathers’ reports [14]. In the current study, fathers’ reports of perceived social support might have been too early in pregnancy to detect an association with child outcomes at 8 years, and future studies should include fathers’ reports on social support in early childhood.

### Strengths and limitations

The population-based cohort design, prospective follow-up, use of parent diagnostic interviews, and mothers’ reports on social support on multiple time points, were among the strengths of the current study. The study also had several limitations. We did not distinguish between social support and loneliness in the analyses. Moreover, perceived social support is measured with various approaches in the literature [6], leaving uncertainty if we have examined the same phenomenon as other studies employing the same term. We included reports of received social support and feelings of loneliness to encompass different elements of the phenomenon. In addition, we set the criterion for being categorized with “low social support” as strict as the items allowed to form a distinct group experiencing apparent low support. Unfortunately, we did not have fathers’ reports on several time points, as we had for mothers’ reports, and we were not able to conclude whether fathers’ perceived social support later in pregnancy and early childhood influenced child symptoms. Children’s symptoms of anxiety and ADHD were reported by mothers only, introducing the risk of shared method variance, as both maternal symptoms and low social support may have influenced the reporting of child symptoms. However, mothers are generally considered a crucial source of information to the clinical assessments of child psychiatric symptoms. A strength of our study is the longitudinal design, with child symptoms measured at ages 3.5 and 8 years, the latter being 5 years after the last maternal self-report. This approach should reduce the risk of same-rater-bias compared to cross-sectional studies. Furthermore, the study was designed to estimate the risk of child anxiety and ADHD symptoms, not to detect causal effects. In particular, we were not able to control for personality traits that are known to be associated with social support, or genetic transmission of shared genetic risk factors between generations. Finally, there were selection biases due to attrition [16, 44]. In the ADHD-study it has previously been reported only slight differences between responders and non-responders, in the mean years of parent education between responders and non-responders, with no significant differences in sex distribution or in number of ADHD symptoms at age 3.5 years [41]. However, the population-based design and enrolment to the MoBa study likely reduced the inclusion of families with substantial mental health issues and low socioeconomic status, as indicated by previous analyses [45]. Consequently, it is reasonable to assume that if these families had participated, the significant associations and trends found in the present study between low social support and child symptoms would have been stronger.

In conclusion, the findings of the present study suggest an association between low maternal social support in pregnancy and early childhood, and subsequent offspring anxiety and ADHD symptoms. Social support constitutes one piece in the intricate interplay of both genetic and environmental factors influencing child symptoms. Although the underlying mechanisms are yet to be clarified, our findings indicate that this association is beyond what can be explained by cooccurring symptoms of maternal anxiety/depression or ADHD, other maternal stressors and child risk factors. Interventions aimed at mobilizing social support to improve mental health in children have shown promise [46]. The present study identified a significant association between low maternal social support and child symptoms of anxiety and ADHD. Although we lack causal evidence, these findings could suggest that directing attention towards mothers with low social support may hold significance for child symptoms years later.

**Table 1:** Descriptive statistics of child and maternal continuous variables for the whole sample, and comparisons between children with mothers reporting low and good social support

	All participants		Low maternal social support ^a^		Good maternal social support ^b^		T	*p*
	*n*	M (SD)	*n*	M (SD)	*n*	M (SD)		
Child symptom count								
Anxiety 3.5 years	781	1.08 (1.66)	90	1.84 (2.26)	691	0.98 (1.53)	-4.68*	< 0.001
Anxiety 8 years	781	2.08 (1.85)	90	2.91 (2.44)	691	1.97 (1.73)	3.55	< 0.001
ADHD 3.5 years	781	3.96 (3.84)	90	4.86 (4.18)	691	3.84 (3.78)	-1.72	0.02
ADHD 8 years	781	2.57 (4.04)	90	4.01 (5.11)	691	2.38 (3.84)	-2.92*	< 0.01
Maternal SCL- scores								
-week 15	764	0.10 (0.30)	87	1.65 (0.69)	677	1.28 (0.38)	-4.19*	< 0.001
-week 30	751	0.05 (0.22)	85	1.55 (0.51)	666	1.27 (0.30)	-5.11*	< 0.001
-18 months	726	0.10 (0.30)	86	1.76 (0.63)	640	1.34 (0.38)	-6.01*	< 0.001
-3 years	765	0.13 (0.34)	88	1.90 (0.76)	677	1.36 (0.41)	-6.45*	< 0.001
Maternal ASRS- score	761	8.06 (3.65)	85	9.63 (4.28)	676	7.86 (3.52)	-3.66*	< 0.001
Maternal education (years)	755	15.31 (2.34)	88	14.69 (2.67)	675	15.39 (2.27)	2.34*	0.02

*Note *^a^ Low social support reported at least once in pregnancy weeks 15 and 30 and at child ages 18 months and 3 years; ^b^ Good social support at all assessments in pregnancy weeks 17 and 30 and at child ages 18 months and 3 years *Equal variances not assumed

The proportions of children categorized in the anxiety group when low/good maternal social support were 30% (*n* = 27) / 12% (*n* = 80) at 3.5 years (χ^2^ = 22.86, *p* < .001), and 23% (*n* = 21) / 10% (*n* = 70) at age 8 years (χ^2^ = 13.48, *p* < .001). The proportions of children categorized in the ADHD group when low/good maternal social support were 24% (*n* = 22) / 17% (*n* = 118) at 3.5 years (χ^2^ = 2.94, *p* = .087), and 23% (*n* = 21) / 7% (*n* = 64) at 8 years (χ^2^ = 16.26, *p* < .001)

**Table 2:** Logistic regression, univariable analyses

	**Child anxiety 3.5 years**				**Child ADHD 3.5 years**			
	**B (SE)**	**OR**	**95% CI**	***p***	**B (SE)**	**OR**	**95% CI**	***p***
Low social support ^a^	1.19 (0.26)	3.27	1.97–5.44	< 0.001	0.45 (0.90)	1.57	0.93–2.64	0.09
Marital status ^b^	0.12 (0.40)	1.13	0.52–2.47	0.76	0.75 (0.32)	2.11	1.14–3.91	0.02
Low paternal social support ^c^	0.19 (0.46)	1.21	0.49–2.99	0.67	0.83 (0.36)	2.29	1.12–4.66	0.02
	**Child anxiety 8 years**				**Child ADHD 8 years**			
	**B (SE)**	**OR**	**95% CI**	***p***	**B (SE)**	**OR**	**95% CI**	***p***
Low social support ^a^	0.99 (0.28)	2.70	1.56–4.67	< 0.001	1.09 (0.28)	2.98	1.72–5.18	< 0.001
Marital status ^b^	0.62 (0.37)	1.86	0.90–3.84	0.09	1.20 (0.34)	3.32	1.72–6.41	< 0.001
Low paternal social support ^c^	0.59 (0.44)	1.80	0.76–4.22	0.18	0.34 (0.50)	1.41	0.53–3.73	0.49

*Note *^a^ Low social support reported at least once in pregnancy weeks 15 and 30 and at child ages 18 months and 3 years; ^b^ Mother living without a partner in pregnancy or divorced/separated/widowed before age 3 years; ^c^ Father’s report of low social support at week 15 in pregnancy

**Table 3:** Logistic regression, multivariable models

	**Child anxiety 3.5 years**				**Child ADHD 3.5 years**			
	**B (SE)**	**OR**	**95% CI**	***p***	**B (SE)**	**OR**	**95% CI**	***p***
Low social support^a^	0.88 (0.31)	2.42	1.32–4.44	< 0.01				
Maternal Anx/Dep^b^	0.90 (0.26)	2.07	1.48–4.07	< 0.001	0.35 (0.24)	1.42	0.89–2.28	0.14
Maternal ADHD^c^								
Marital status^d^								
Low paternal social support^e^					0.70 (0.39)	2.02	0.95–4.29	0.07
Parental education^f^					0.73 (0.24)	2.08	1.29–3.35	< 0.01
*Constant*	*-2.32 (0.15)*	*0.10*		*< 0.001*	*-1.89 (0.14)*	*0.15*		*< 0.001*
	**Child anxiety 8 years**				**Child ADHD 8 years**			
	**B (SE)**	**OR**	**95% CI**	***p***	**B (SE)**	**OR**	**95% CI**	***p***
Low social support^a^	0.65 (0.34)	1.92	0.99–3.73	0.06	0.68 (0.36)	1.98	0.98–3.99	0.06
Maternal Anx/Dep^b^	0.71 (0.29)	2.04	1.16–3.56	0.01				
Maternal ADHD^c^	0.81 (0.39)	2.25	1.04–4.86	0.04	0.63 (0.45)	1.98	0.80–4.42	0.15
Marital status^d^								
Low paternal social support^e^					0.68 (0.45)	1.98	0.83–4.73	0.13
Parental education^f^	0.76 (0.28)	2.14	1.23–3.70	< 0.01	0.86 (0.30)	2.37	1.32–4.27	< 0.01
*Constant*	*-2.66 (0.18)*	*0.07*		*< 0.001*	*-2.72 (0.18)*	*0.07*		*< 0.001*

*Note *^a^ Low social support reported at least once in pregnancy weeks 15 and 30 and at child age 18 months and 3 years; ^b^ Maternal SCL- score ≥2 reported at least once in pregnancy weeks 15 and 30 and at child age 18 months and 3 years; ^c^ ASRS- scores ≥ at 3 years; ^d^ Mother living without a partner in pregnancy, or divorced/separated/widowed before age 3 years; ^e^ Father’s report of low social support at week 15 in pregnancy; ^f^ Mean maternal and paternal education length ≥ 12 years

## Electronic supplementary material

Below is the link to the electronic supplementary material.

Supplementary Material 1

## Data Availability

The data that support the findings of this study were available from MoBa at the Norwegian Institute of Public Health, but restrictions apply to the availability of these data, used under license for the current study, and so are not publicly available.
