# Supplementary material for: Low perceived social support in mothers during pregnancy and early childhood; associations with anxiety and ADHD symptoms in children at 3 and 8 years
Source: Soc Psychiatry Psychiatr Epidemiol. 2024 Nov 6;60(4):895–903. doi: 10.1007/s00127-024-02792-1 (PMC12031961; doi:10.1007/s00127-024-02792-1)
Supplement: Supplementary file 1 — Supplementary Material 1 [file 127_2024_2792_MOESM1_ESM.pdf]

Supplemental Figure 1. Enrolment and attrition flowchart

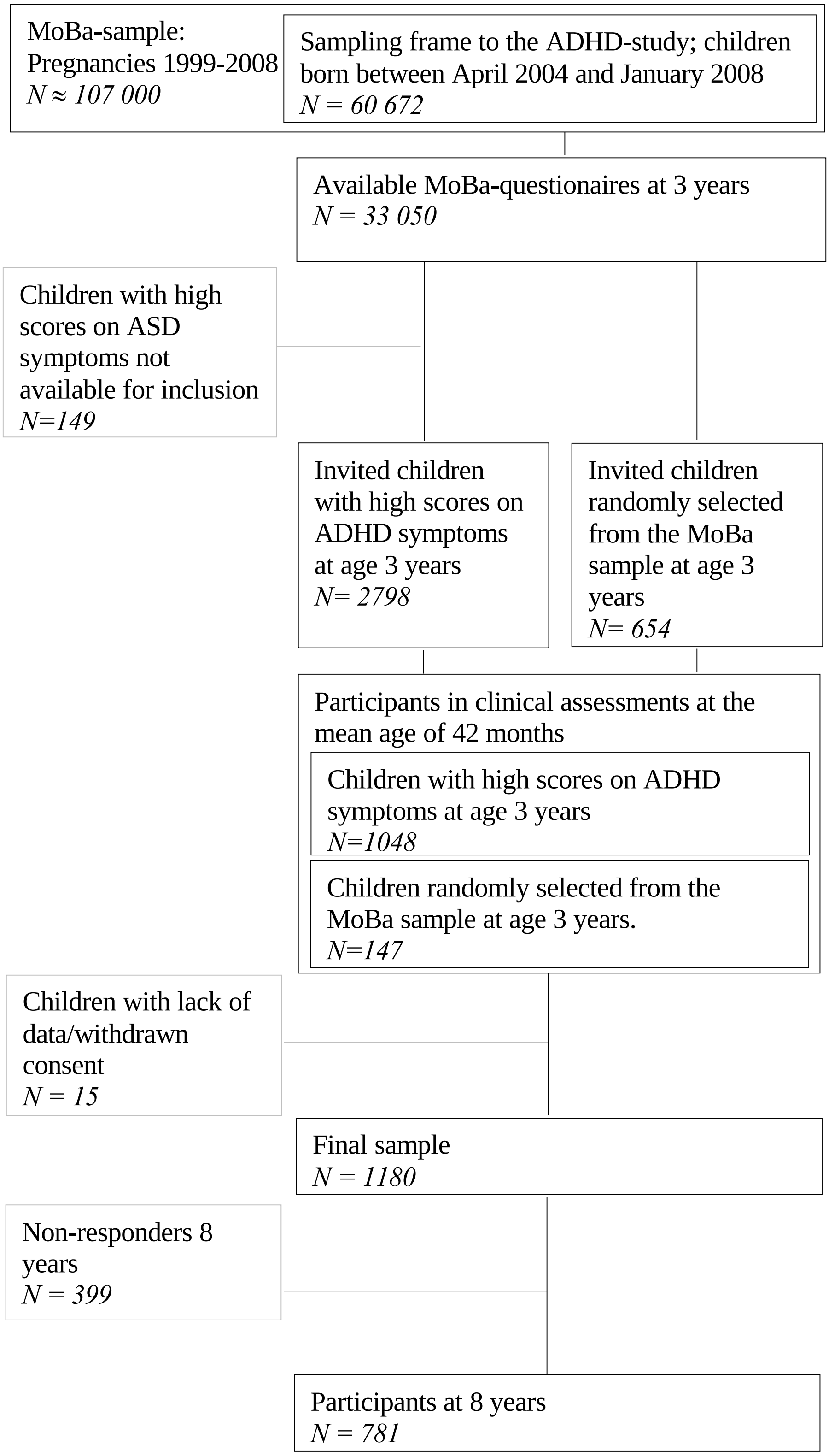

Note: MoBa, The Norwegian Mother, Father and Child Study; ADHD, attention-deficit/hyperactivity disorder; ASD, autism spectrum disorder.

Supplemental Table 1. Measurements and number of participants at the different timepoints

	Pregnancy		Child age			
	Week 15	Week 30	18 months	3 years	3.5 years	8 years
**Child anxiety and ADHD (*n*)**					PAPA interview (*781*)	CSI-4 (*781*)
**Maternal social support (*n*)**	Items from MoBa (*765*)	Items from MoBa (*746*)	Items from MoBa (*728)*	Items from MoBa *(755*)		
**Maternal anxiety and depression (*n*)**	SCL-5 in MoBa (*764*)	SCL-8 in MoBa (*751*)	SCL-8 in MoBa (*726*)	SCL-8 in MoBa (*765*)		
**Maternal ADHD (*n*)**				ASRS-6 in MoBa (*761*)		

Note: ADHD, attention-deficit/hyperactivity disorder; SCL, short versions of the Hopkins Symptom Checklist; ASRS-6, the Adult Self-Report Scale-6 for attention-deficit/hyperactivity disorder; PAPA interview, Preschool Age Psychiatric Assessment; SCI-4, Child Symptom Inventory-4.
